# A new model for the biodegradation kinetics of oil droplets: application to the Deepwater Horizon oil spill in the Gulf of Mexico

**DOI:** 10.1186/1467-4866-14-4

**Published:** 2013-10-20

**Authors:** Javier Vilcáez, Li Li, Susan S Hubbard

**Affiliations:** 1 John and Willie Leone Family Department of Energy and Mineral Engineering, The Pennsylvania State University, University Park, PA 16802, USA; 2 EMS Energy Institute, The Pennsylvania State University, University Park, PA 16802, USA; 3 Earth and Environmental Systems Institute (EESI), The Pennsylvania State University, University Park, PA 16802, USA; 4 Earth Sciences Division, Lawrence Berkeley National Laboratory, Berkeley, CA 94720, USA; 5 Currently at the University of Tokyo, Tokyo, Japan

**Keywords:** Modeling, Biodegradation, Oil droplets, Size distribution, Shrinking core model, Gulf of Mexico oil spill, Deepwater horizon oil spill

## Abstract

Oil biodegradation by native bacteria is one of the most important natural processes that can attenuate the environmental impacts of marine oil spills. Existing models for oil biodegradation kinetics are mostly for dissolved oil. This work developed a new mathematical model for the biodegradation of oil droplets and applied the model to estimate the time scale for oil biodegradation under conditions relevant to the Deepwater Horizon oil spill in the Gulf of Mexico. In the model, oil is composed of droplets of various sizes following the gamma function distribution. Each oil droplet shrinks during the microbe-mediated degradation at the oil-water interface. Using our developed model, we find that the degradation of oil droplets typically goes through two stages. The first stage is characterized by microbial activity unlimited by oil-water interface with higher biodegradation rates than that of the dissolved oil. The second stage is governed by the availability of the oil-water interface, which results in much slower rates than that of soluble oil. As a result, compared to that of the dissolved oil, the degradation of oil droplets typically starts faster and then quickly slows down, ultimately reaching a smaller percentage of degraded oil in longer time. The availability of the water-oil interface plays a key role in determining the rates and extent of degradation. We find that several parameters control biodegradation rates, including size distribution of oil droplets, initial microbial concentrations, initial oil concentration and composition. Under conditions relevant to the Deepwater Horizon spill, we find that the size distribution of oil droplets (mean and coefficient of variance) is the most important parameter because it determines the availability of the oil-water interface. Smaller oil droplets with larger variance leads to faster and larger extent of degradation. The developed model will be useful for evaluating transport and fate of spilled oil, different remediation strategies, and risk assessment.

## Background

Oil spills can cause serious environmental problems and ecological consequences. The Deepwater Horizon oil spill in the Gulf of Mexico occurred in April 2010 is a recent example. This spill led to the accidental release of over 4.9 million barrels of oil [1] at a depth of 1500 m [2] below the water surface. After and during the oil spill it is a common practice to introduce chemical dispersants near the spill region. Under these conditions, spilled oil can not only dissolve in sea water, but also form oil droplets of various sizes. Although large oil droplets can rise to the sea surface due to the buoyancy effect, previous studies suggest that small oil droplets would remain underwater [3-5]. As such, spilled oil can exist in both dissolved form and as oil droplets in deep water.

Spilled oil is subject to various natural attenuation processes, including, for example, mixing, dilution, transport through advection with the sea water currents [6], dissolution, evaporation, and biodegradation [7]. Among these, biodegradation can play a major role in ultimately transforming the spilled oil. In marine environments, many oil degrading microorganisms can use oil as their electron and carbon source and oxygen as their electron acceptor to ultimately degrade oil to carbon dioxide [8-10].

With documentation that spilled oil can occur in tiny droplets in deep water and that natural biodegradation can indeed occur, it is critical to estimate how fast oil droplets can be biodegraded. Oil is in general a complex mixture of various organic compounds, including chained and aromatic hydrocarbons, which can differ significantly in their biodegradation kinetics [11]. In addition, the biodegradation kinetics can also be affected by the initial oil concentration, the abundance of oil degrading microbe, the concentration of dissolved oxygen, and the availability of the oil-water interface. It has been reported that the greater the oil-water interface, the faster the oil degradation by microbes [9,12,13]. As such, their size distribution can play a critical role in determining the biodegradation kinetics of oil spills.

Various models have been proposed to quantify the biodegradation kinetics of spilled oil. The majority of existing models assume that spilled oil is in soluble form [14-16]. Few biodegradation models have also been proposed to take into account the presence of oil droplets [17-19]. Among these, oil droplets have been assumed of uniform size with the presence of abundant oil degrading microbes, which is not applicable for most cases. For instance, in the Gulf of Mexico oil spill, the high pressure at 1500 m underwater and the addition of a chemical dispersant immediately broke the oil into tiny oil droplets of various sizes not much bigger than the size of a microbe (1 μm approximately), with typical mean oil droplet size between 20 – 30 μm [20,21]. The background oil degrading microbe concentration was approximately 2.73 × 10^4^ cells/ml [7]. The oil concentration was approximately 0.4 mg/L [2]. If all 0.4 mg/L of oil was assumed to exist in the form of 20 μm diameter oil droplets, the oil droplets concentration is approximately 1 × 10^5^ droplets/ml. There is a significant lack of understanding on the oil degradation under these conditions where oil is highly dispersed with small oil droplets.

The goal of this study is to assess the biodegradation rates of dispersed oil droplets of mixed composition through developing and implementing a new mathematical model. The model takes into account the size distribution of oil droplets, microbial activity as a function of the available oil-water interface, as well as the shrinking and conversion process of oil droplets. We used the developed model to examine how various factors affect the time scale of oil droplet biodegradation, including the droplet size distribution, initial oil and microbial concentration, maximum microbial density at the water-oil interface, and the chemical composition of the oil droplets.

## Mathematical model

### Conceptual model of the system

**Figure 1:**
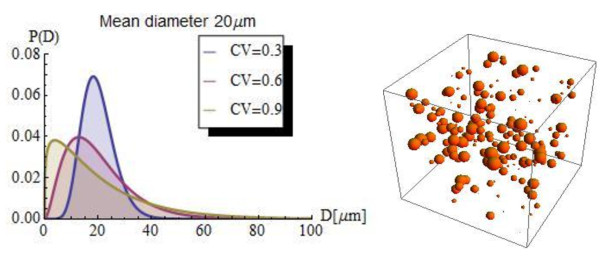
**Size distribution and conceptual representation of oil droplets.** Left: Gamma function of oil droplet size distribution for various coefficients of variation (CV). Right: Schematic representation of dispersed oil droplets in the control volume. The formulated shrinking oildroplet model is for a control volume containing dispersed oil droplets with their sizes following a gamma distribution function.

Conceptually, a control volume includes the electron acceptor oxygen, oil degrading bacteria, and oil droplets. The oil droplets have a size distribution of the gamma function. Biodegradation of oil in this study implies shrinkage of oil droplets by microbes attached to the droplet surface (Figure 1), which consumes oxygen and oil while at the same time produces carbon dioxide and water. According to observations in the oil spill of the Gulf of Mexico, the concentration of oxygen can remain fairly constant without substantial drawdown [2,7]. This is probably because of the relatively low concentration of spilled oil in the sea water that is typically within the range of 0.1 – 1.0 mg/L. With typical original dissolved oxygen concentration between 3.5 – 4.0 mg/L, even the complete biodegradation of oil will not deplete oxygen. As such, it is assumed that oxygen does not control the biodegradation and a slight decrease in oxygen concentration does not affect the biodegradation rate of spilled oil in sea water.

In the case of dissolved oil biodegradation, the amount of active oil degrading bacteria are simply the microbial concentration times the total volume of the bulk fluid. For the oil droplets, however, the total amount of active biodegrading microbe depends on the microbial density at the water-oil interface. Because the average size of a microbe is around 1.0 μm, for most calculations the maximum microbial density on the oil droplet is assumed to be equal to 1 cell/μm^2^[8]. We do vary this value between 1.0 and 10.0 cell/μm^2^ in the sensitivity analysis to understand its impact on the time scale of degradation. The total amount of acting bacteria is then the available water-oil interface area times the maximum microbial density. In this study, the term oil is used to describe the total petroleum hydrocarbons (TPH) regardless of its composition. Oil droplets are assumed to be made up of selected model compounds which are known to persist in the environment due to their recalcitrant nature. These chemicals are listed in Table 1 along with their biodegradation kinetic coefficients estimated from biodegradation measurements of pure samples.

**Table 1:** Kinetic parameters for representative hydrocarbon compounds

**Compound**	**μ (h**^**-1**^**)**	**K**_**s**_**(g-oil/m**^**3**^**)**	**Y**_**oil**_**(g-cell/g-oil)**	**Reference**
Naphthalene (C_10_H_8_)	0.256	0.57	0.4	[26]
1-Methylnaphthalene (C_11_H_10_)	0.310	5.3	0.5	[26]
2-Methylnaphthalene (C_11_H_10_)	0.240	3.1	0.4	[26]
2-Ethylnaphthalene (C_12_H_12_)	0.240	2.7	0.4	[26]
Phenanthrene (C_14_H_10_)	0.240	2.2	0.4	[26]
Anthracene (C_14_H_10_)	0.240	2.5	0.4	[26]
Pyrene (C_16_H_10_)	0.240	0.69	0.4	[26]
Benzene (C_6_H_6_)	0.335	3.17	1.04	[41]
Toluene (C_7_H_8_)	0.543	1.96	1.22	[41]
Xylene (C_8_H_10_)	0.535	4.55	0.25	[41]

### Oil biodegradation reactions

Oil is composed of a broad family of hundreds of chemical compounds. The calculation of biodegradation rates of the spilled oil requires information on the stoichiometric coefficients such as the mass of biodegraded hydrocarbon compounds per oxygen consumed (*h*) and the microbial biomass produced per mass of hydrocarbon compound biodegraded (Y_oil_) for individual or group of hydrocarbon. Values of Y_oil_ have been experimentally determined for various hydrocarbon compounds. To explicitly model also the amount of oxygen consumed, values of *h* were calculated based on the principle of energetics and substrate partition into energy production and cell synthesis. With the general elemental composition of bacteria represented by the formula C_5_H_7_O_2_N, the half-reaction of cell synthesis can be written as follows [22]:

(1) $$\begin{array}{ll} \;\frac{1}{5}{\text{CO}}_{2} & +\frac{1}{20}{\text{NH}}_{4}^{+}+\frac{1}{20}{\text{HCO}}_{3}^{-}+H^{+}+e^{-}\to \frac{1}{20}C_{5}H_{7}O_{2}N\\ & +\frac{9}{20}H_{2}O \end{array}$$

where the electron equivalents per mol of cell is 20e^-^. Therefore, the fraction of donor electrons synthetized into new biomass (f_s_) can be calculated from the following expression:

(2) $$\begin{array}{ll} \;f_{s}\left(\frac{e^{-}-\mathrm{cells}}{e^{-}-C_{x}H_{y}}\right) & =Y_{\text{oil}}\left(\frac{\text{mol}-\text{cell}}{\text{mol}-C_{x}H_{y}}\right)\left(\frac{{20e}^{-}-\text{cell}}{\text{mol}-\text{cell}}\right)\\ & \;\left(\frac{\text{mol}-C_{x}H_{y}}{{\text{ne}}^{-}-C_{x}H_{y}}\right) \end{array}$$

where ne^-^ is the number of donor electrons per mol of biodegraded hydrocarbon compound (C_x_H_y_). Values of ne^-^ were obtained from the half-reaction of C_x_H_y_ conversion to CO_2_. The half-reactions of selected hydrocarbon compounds along with their corresponding values of ne^-^ are listed in Table 2. Values of f_s_ for each individual hydrocarbon compound were then used to calculate the mass of biodegraded hydrocarbon compounds per oxygen consumed (*h*):

(3) $$h=\left({1‒f}_{s}\right)\;a$$

where *a* is the mass of oxygen consumed per mass of hydrocarbon converted to carbon dioxide when microbial biomass is not synthesized (Table 3). Table 4 summarizes values of *h* for individual hydrocarbons. These values are consistent with the thermodynamics of bacteria energetics and carbon balance which states that with the same electron acceptor, the yield coefficient should be similar for each unit carbon [23-25].

**Table 2:** Half reactions for individual hydrocarbon compounds

**Compound**	**Reaction**	**ne**^**-**^
Naphthalene (C_10_H_8_)	C_10_H_8_ + 20H_2_O → 10CO_2_ + 48H^+^ + 48e^-^	48
1-Methylnaphthalene (C_11_H_10_)	C_11_H_10_ + 22H_2_O → 11CO_2_ + 54H^+^ + 54e^-^	54
2-Methylnaphthalene (C_11_H_10_)	C_11_H_10_ + 22H_2_O → 11CO_2_ + 54H^+^ + 54e^-^	54
2-Ethylnaphtalene (C_12_H_12_)	C_12_H_12_ + 24H_2_O → 12CO_2_ + 60H^+^ + 60e^-^	60
Phenanthrene (C_14_H_10_)	C_14_H_10_ + 28H_2_O → 14CO_2_ + 66H^+^ + 66e^-^	56
Anthracene (C_14_H_10_)	C_14_H_10_ + 28H_2_O → 14CO_2_ + 66H^+^ + 66e^-^	56
Pyrene (C_16_H_10_)	C_16_H_10_ + 32H_2_O → + 16CO_2_ + 74H^+^ + 74e^-^	74
Benzene (C_6_H_6_)	C_6_H_6_ + 12H_2_O → + 6CO_2_ + 30H^+^ + 30e^-^	30
Toluene (C_7_H_8_)	C_7_H_8_ + 14H_2_O → + 7CO_2_ + 36H^+^ + 36e^-^	36
Xylene (C_8_H_10_)	C_8_H_10_ + 16H_2_O → + 8CO_2_ + 42H^+^ + 42e^-^	42

**Table 3:** Oxidation reactions of hydrocarbon compounds without including microbial growth

**Compound**	**Reaction**
Naphthalene (C_10_H_8_)	C_10_H_8_ + 12O_2_ → 4H_2_O + 10CO_2_
1-Methylnaphthalene (C_11_H_10_)	C_11_H_10_ + 13.5O_2_ → 5H_2_O + 11CO_2_
2-Methylnaphthalene (C_11_H_10_)	C_11_H_10_ + 13.5O_2_ → 5H_2_O + 11CO_2_
2-Ethylnaphtalene (C_12_H_12_)	C_12_H_12_ + 14O_2_ → 6H_2_O + 12CO_2_
Phenanthrene (C_14_H_10_)	C_14_H_10_ + 16.5O_2_ → 5H_2_O + 14CO_2_
Anthracene (C_14_H_10_)	C_14_H_10_ + 16.5O_2_ → 5H_2_O + 14CO_2_
Pyrene (C_16_H_10_)	C_16_H_10_ + 18.5O_2_ → 5H_2_O + 16CO_2_
Benzene (C_6_H_6_)	C_6_H_6_ + 7.5O_2_ → 3H_2_O + 6CO_2_
Toluene (C_7_H_8_)	C_7_H_8_ + 9O_2_ → 4H_2_O + 7CO_2_
Xylene (C_8_H_10_)	C_8_H_10_ + 10.5O_2_ → 5H_2_O + 8CO_2_

**Table 4:** Calculated coefficients for individual hydrocarbons

**Compound**	**MW (g/mol)**	**Y**_**oil**_**(mol-cell/mol-oil)**	**f**_**s**_**(e**^**-**^**-cell/ e**^**-**^**-C**_**x**_**H**_**y**_**)**	**h (mol-O**_**2**_**/mol-oil)**
Naphthalene (C_10_H_8_)	128	0.4531	0.1888	9.7345
1-Methylnaphthalene (C_11_H_10_)	142	0.628	0.2326	10.3599
2-Methylnaphthalene (C_11_H_10_)	142	0.439	0.1626	11.3049
2-Ethylnaphtalene (C_12_H_12_)	156	0.552	0.1840	11.424
Phenanthrene (C_14_H_10_)	178	0.6300	0.2250	12.7875
Anthracene (C_14_H_10_)	178	0.6300	0.2250	12.7875
Pyrene (C_16_H_10_)	202	0.7150	0.1932	14.925
Benzene (C_6_H_6_)	78	0.7178	0.4785	3.911
Toluene (C_7_H_8_)	92	0.9933	0.5518	4.0335
Xylene (C_8_H_10_)	106	0.2345	0.1147	9.3275

Molecular weight of bacterial cells is 113 based on the formula C_5_H_7_O_2_N.

### Biodegradation kinetics for dissolved oil

Because oil is made up of various types of hydrocarbons of different biodegradability, the biodegradation of oil is usually represented by a multi-substrate Monod model where multiple growth substrates are available to oil degrading microbes [26,27]. In attempting to formulate the theoretical work here we first tried both multi-substrate and sole-substrate models for the degradation of soluble oil. The predicted results by the two models were very similar (not shown here). Because the kinetic coefficients for sole-substrate models are typically more available from total petroleum hydrocarbons (TPH) biodegradation measurements [28] or oxygen uptake measurements [29] than those for multi-substrate models, for simplicity here we chose to use the sole-substrate model by categorizing different groups of hydrocarbon into different pseudo-components. The biodegradation rate of each pseudo-component is then represented by a sole-substrate Monod’s model [30]:

(4) $$r_{b}=\mu_{\text{max}}\frac{C_{\text{oil}}}{K_{s}+C_{\text{oil}}}B$$

(5) $$r_{\text{oil}}=\frac{1}{Y_{\text{oil}}}r_{b}$$

(6) $$r_{O_{2}}=\frac{1}{Y_{O_{2}}}r_{b}$$

where r_b_ is the rate of microbial growth (cells/L - h), r_oil_ and $r_{O_{2}}$ are the rates of oil degradation (mg-oil/L - h) and oxygen consumption (mg-O_2_/L - h), respectively, μ_max_ is the maximum rate coefficient (h^-1^), C_oil_ is the total oil concentration (mg/L), K_s_ is the half saturation constant (mg/L), and B is the concentration of microbes (cells/L) in the bulk fluid. The Monod equation is widely used to describe microbial growth and substrate consumption [22]. The Monod parameters here, including μ_max_ and K_s_, can represent the rate parameters not only due to regular oil biodegradation, but also those affected by other degrading mechanisms such as co-metabolism [31,32]. In this case, the degradation of the co-metabolized compound should still follow the degradation kinetics of the primary compound. Therefore, the effects of co-metabolism will be reflected in the values of these parameters however will not change the general form of the formulation.

By using the sole-substrate model, it is assumed that there are no interactions, including inhibition, among different substrates. This represents one of the simplifications of the model, the validity of which may need to be determined by experimental work in the future. Ideally, the rate equation should include a term to represent the microbial decay. Although its incorporation in the kinetic model is straightforward, it is not done here because experimental data on the decay rate of oil degrading microbes at oil concentration levels relevant to marine oil spills are yet to be available. As such, the rates of bacterial growth here represent rates under relatively optimum conditions.

To take into account the different biodegradability of individual hydrocarbon compounds in each pseudo-component, the oxygen demand and kinetic coefficients of Eq. (4, 5, 6) were calculated based on the composition of the pseudo-component using the following expressions:

(7) $$Y_{O_{2}}=\overset{n}{\underset{i=1}{\sum }}x_{i}Y_{O_{2},i}$$

(8) $$\mu_{\max}=\overset{n}{\underset{i=1}{\sum }}x_{i}\mu_{\max,}{}^{i}$$

(9) $$K_{s}=\overset{n}{\underset{i=1}{\sum }}x_{i}K_{\text{s,i}}$$

where *Y*_*O2*_ is the microbial biomass produced per mass of oxygen consumed from the biodegradation of hydrocarbon type *i*, and *x*_*i*_ is the mass fraction of the hydrocarbon type *i*. All other terms with the subscript *i* represent the corresponding parameters for each individual hydrocarbon *i*. Table 1 summarizes kinetic parameters for representative recalcitrant hydrocarbons. The yield coefficient for oxygen is usually not reported. Therefore, its value has been estimated following the procedure described in previous sections.

### The shrinking-core model (SCM) for the biodegradation of one oil droplet

The kinetics of particle size reduction due to reactions at the water-particle interface is usually represented by the shrinking core model (SCM) [33]. The rate of particle size reduction can potentially be controlled by both the rate of chemical transport to and away from the particle surface and the reaction rate at the particle surface. In this work, the reaction rate at the droplet surface was assumed to control the biodegradation process because of the oxygen abundance and the tendency of microbes to attach to droplet surface. Under this condition, based on the unit surface of unreacted oil droplets, an expression for the calculation of the biodegradation rate can be formulated in terms of the rate of oxygen consumption:

(10) $$-\frac{1}{S}\frac{{\text{dN}}_{\text{oil}}}{\text{dt}}=-\frac{h}{S}\frac{{\text{dN}}_{O_{2}}}{\text{dt}}$$

where S is the total surface area of oil droplets (μm^2^), N_oil_ is the mass of oil, and N_O2_ is mass of oxygen. Here the rate of oxygen consumption per unit surface is represented by the Monod’s equation:

(11) $$-\frac{1}{S}\frac{{\text{dN}}_{O_{2}}}{\text{dt}}=\frac{\mu_{\text{max}}}{Y_{O_{2}}}\frac{C_{\text{oil}}}{K_{s}+C_{\text{oil}}}B_{s}$$

here B_s_ is the concentration of microbes at the oil droplet surface.

The mass of oil (N_oil_) can be written in terms of the oil droplet diameter and density:

(12) $${\text{dN}}_{\text{oil}}=\rho \frac{\pi }{2}D^{2}\text{dD}$$

where D is the oil droplet diameter and ρ is the oil density. By substituting Eqs. (11) and (12) into Eq. (10) and taking into account the fact that Y_oil_ = *h*Y_O2_ and S = πD^2^, the change rate of oil droplet diameter as a function of bacterial concentration at the oil droplet surface can be expressed as follows:

(13) $$-\frac{\rho }{2}\frac{\text{dD}}{\text{dt}}=\frac{\mu_{\text{max}}}{Y_{\text{oil}}}\frac{C_{\text{oil}}}{K_{s}+C_{\text{oil}}}B_{s}$$

Integration of Eq. (13) gives the fraction of the biodegraded oil droplet volume (X_1-droplet_) as a function of time [30]:

(14) $$1-{\left(1-X_{1‒\text{droplet}}t\right)}^{1/3}=\frac{k_{\text{rn}}}{D}t$$

where

(15) $$k_{\text{rn}}=\frac{2}{\rho_{\text{oil}}}\frac{\mu_{\text{max}}}{Y_{\text{oil}}}\frac{C_{\text{oil}}}{K_{s}+C_{\text{oil}}}B_{s}$$

The term X_1-droplet_ quantifies the fraction of oil conversion into CO_2_ for a single oil droplet.

### The model for a distribution of oil droplets with varying size

The fraction of biodegraded oil for a distribution of oil droplets (X) is a function of the oil droplet size distribution. Therefore, Eq. (15) should be integrated to incorporate the effects of droplet size distribution. This has been done for instance by Gbor and Jia [34] and by McIlvried and Masstoh [35]. For a surface reaction-controlled process, the overall conversion of oil droplets of various sizes is found by integrating Eq. (15) with respect to the size of oil droplets (D):

(16) $$X=1-\overset{\infty }{\underset{0}{\int }}{\left(\frac{1‒k_{\text{rn}}t}{D}\right)}^{3}P\left(D\right)\text{dD}$$

Here P(D) is the oil droplet size distribution function.

As reaction proceeds, a time is reached when the smallest particle in the distribution is completely biodegraded. This is represented by including a variable integration limit D_t_:

(17) $$X=1-\overset{D_{t}}{\underset{0}{\int }}0P\left(D\right)\text{dD}-\overset{D_{\text{max}}}{\underset{D_{t}}{\int }}{\left(1-\frac{k_{\text{rn}}t}{D}\right)}^{3}P\left(D\right)\text{dD}$$

where all oil droplets of size less than D_t_ are fully reacted and thus have a conversion value of 1, and D_max_ is the size of the largest oil droplet in the control volume. The application of this condition to Eq. (16) gives the expression to calculate D_t_ at any time t:

(18) $$D_{t}=k_{\text{rn}}t$$

where oil droplets of size greater than D_t_ are partially reacted.

In this study a gamma particle size distribution function is used for P(D) because this function has been reported in studies describing the effect of chemical dispersants on oil droplet size distribution. This is also consistent with the oil droplet size reported for the oil spill [4,36]. The gamma function is given by:

(19) $$P\left(D\right)=\frac{1}{\beta^{\alpha }\Gamma \left(\alpha \right)}D^{\alpha -1}e^{-D/\beta }$$

where μ = αβ is the mean diameter, σ = α^0.5^β is the standard deviation, and CV = σ/μ = 1/α^0.5^ is the coefficient of variation. Substituting Eq. (19) into Eq. (18) gives the overall volume fraction of the biodegraded oil droplets over the total initial oil with a gamma size distribution:

(20) $$X=1-\int_{D_{t}}^{D_{\text{max}}}\left(1-\frac{k_{\text{rn}}t}{D}\right)\frac{1}{\beta^{\alpha }\Gamma \left(\alpha \right)}D^{\alpha -1}e^{-D/\beta }\text{dD}$$

### Concentration of microbes on the water-oil interface

The water-oil interfacial area is an important parameter that controls the oil droplet surface availability for the colonization of microbes. The specific interfacial area *A* (the ratio of the total surface area over the total volume of all droplets) is given by:

(21) $$A=\frac{\pi \sum_{j}n_{j}D_{j}^{2}}{\pi \sum_{j}n_{j}D_{j}^{3}/6}$$

where *n*_*j*_ is the number of oil droplets of the same diameter (D_j_). The total interfacial area (S) in the control volume is obtained by integrating equation (21) over the control volume (V). Assuming that oil droplets are uniform in the whole control volume: S = 6V_o_/ds, where the Sauter mean droplet diameter (ds) for the shrinking droplets is given by:

(22) $$\text{ds}=\frac{\sum n_{j}D_{j}^{3}}{\sum n_{j}D_{j}^{2}}=\frac{\overset{D_{\text{max}}}{\underset{D_{0}}{\int }}{P\left(D\right)D}^{3}\text{dD}}{\overset{D_{\text{max}}}{\underset{D_{0}}{\int }}{P\left(D\right)D}^{2}\text{dD}}\left(1-X\right)$$

Then, the number of cells per unit surface area of droplet is given by:

(23) $$B_{s}=\frac{\text{BV}}{{6V}_{o}}\text{ds}$$

where V is the control volume and V_o_ the volume of oil in the control volume. Because the biodegradation of oil droplets is assumed to take place at the water-oil interface, the accumulation rate of oil degrading microbes in the control volume is given as a function of the microbe concentration at the oil surface:

(24) $$\frac{V}{S}\frac{\text{dB}}{\text{dt}}=\mu_{\text{max}}\frac{C_{\text{oil}}}{K_{s}+C_{\text{oil}}}B_{s}$$

## Results

### Controlling parameters on biodegradation kinetics

In microcosm experiments, a typical oil concentration is in the order of tens of mg/L. In marine oil spills like in the Gulf of Mexico, reported oil concentrations are between 0.1 to 1.0 mg/L [2,7]. Oil droplet size and its distribution have been reported to vary significantly at different sampling points [4]. Furthermore, concentrations of biodegrading microbes can differ extensively depending on the location of the spill. The goal of this section is to use the formulated model to assess the biodegradation time scale and its sensitivity to various factors, including initial oil and microbe concentration, maximum microbial density on oil droplets, oil droplet size distribution, and oil composition. For comparison, we also show the biodegradation kinetics of dissolved oil. The simultaneous biodegradation of dissolved and dispersed oil droplets are not included in this work. Except for the evaluation of the effect of chemical composition, the composition of dissolved oil and dispersed oil droplets in mole fraction used for calculations in all cases is 0.2 for Naphthalene, 0.2 for 1-Methylnaphthalene, 0.1 for 2-Methylnaphthalene, 0.1 for 2-Ethylnaphthalene, 0.1 for Phenanthrene, 0.1 for Anthracene, 0.05 for Pyrene, 0.05 for Benzene, 0.05 for Toluene, and 0.05 for Xylene.

### Biodegradation of dissolved oil

**Figure 2:**
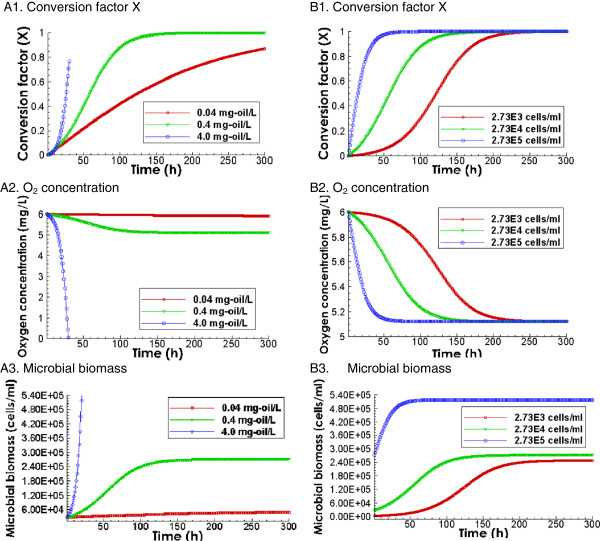
**Predicted evolution of conversion factor X, oxygen concentration, and microbial growth for dissolved oil.** Left column: Effects of initial oil concentrations (0.04, 0.4, and 4.0 mg/L) with the same initial microbe concentration of 2.73 × 10^4^ cells/ml. Right column: Effects of initial microbial concentrations (2.73 × 10^2^, 2.73 × 10^3^, and 2.73 × 10^4^) with the initial dissolved oil concentration of 0.4 mg/L.

Figure 2 shows the total conversion of dissolved oil, oxygen concentration, and microbial concentration calculated using the pseudo-component Monod’s model for three different initial dissolved oil concentrations with initial microbe concentration of 2.73 × 10^4^ cells/ml (left column), and for three initial microbial concentrations with dissolved oil concentration of 0.4 mg/L (right column). The measured concentrations were 0.4 mg/L for oil and 2.73 × 10^4^ cells/ml for oil degrading microbes for the Deepwater Horizon oil spills in deep sea. With 2.73 × 10^4^ cells/ml, the biodegradation rates are larger with larger initial oil concentrations. For instance, with 4.0 mg/L, the rates are highest (Figure 2A1), although the biodegradation stopped at conversion fraction of 0.8 when the oxygen was depleted (Figure 2A2), At 0.4 mg/L, the rates are smaller than with 4.0 mg/L, however here all oil was degraded to depletion at around 150 hours (Figure 2A1). It is interesting to note that with even lower oil concentration of 0.04 mg/L, the degradation rate was lower and the depletion took much longer time (Figure 2A1). This is because the increase of microbial concentration in the bulk fluid is directly proportional to the amount of oil conversion. In the case of initial oil concentration of 0.04 mg/L, the microbial concentration does not increase as much as in the case with 0.4 mg/L, as shown in Figure 2A3. In the cases with the same initial oil concentration of 0.4 mg/L while differing initial microbial concentrations differ (right column), biodegradation rates are much faster with higher initial microbial concentration. At 2.73 × 10^5^ cells/ml, the oil is completely degraded within 40 hours (Figure 2B1). In contrast, at lower microbial concentrations, oil is not depleted until about 150 and 200 hours. This indicates that both initial oil and microbial concentrations can have large impacts on the degradation of dissolved oil. With 0.4 mg/L, all dissolved oil is 100% depleted at the three different initial microbial concentrations, as shown in Figure 2B1. As we will show later, this is not true for the biodegradation of oil droplets.

It is important to note that oxygen uptake under conditions relevant to oil spills in marine environments does not result in high levels of oxygen depletion [2]. Assuming that the initial oxygen concentration is 6 mg/L, oxygen levels after complete biodegradation of 0.4 mg/L of spilled oil with the given composition (Figure 2B2) will not go lower than 5 mg/L. As such, we confirm that the effect of oxygen concentration in the biodegradation kinetics of spilled oil in marine environments can be assumed negligible.

### Effect of initial oil concentration

**Figure 3:**
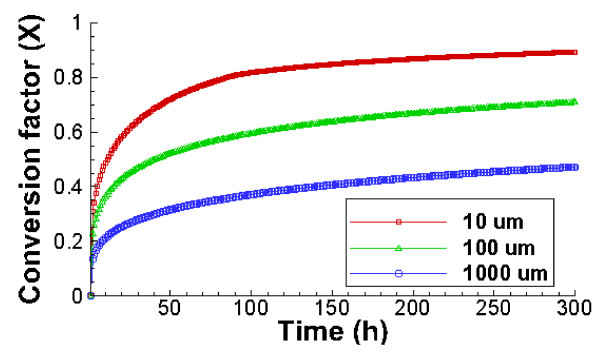
**Predicted evolution of conversion factor X at different initial oil concentrations of dispersed oil droplets.** The initial microbial concentration is 2.73 × 10^4^ cells/ml, the mean diameter of oil droplets is 100 μm, and the CV value is 0.8. Compared to the dissolved oil degradation in Figure 2, the early biodegradation is much faster while the later biodegradation rate is much slower.

Here we quantify the effect of initial oil droplet concentrations on the time evolution of the conversion factor X. Figure 3 compares the biodegradation at the dispersed oil droplet concentrations of 0.04, 0.4 and 4.0 mg/L at the initial bacterial concentration of 2.73 × 10^4^ cells/ml. With other parameters being the same, the biodegradation rate is faster with higher initial dispersed oil droplet concentrations. With approximately 10 days, the conversion factors are 10%, 50%, and 80% for 0.04, 0.4, and 4.0 mg/L, respectively. Compared to that of the dissolved oil, the degradation of the oil droplets starts at higher rates however levels off at later times. The overall conversion factor for the dissolved oil and oil droplets is similar in the 4.0 mg/L case, because the rates of dissolved oil were also limited by available oxygen. However, in the 0.04 and 0.4 mg/L cases, the conversion factors are much lower than that in the dissolved oil. In the case of 0.4 mg/L, only 55% is degraded within 300 hours, in contrast to the complete depletion within 130 hours in the corresponding case of dissolved oil shown in Figure 2A1. The biodegradation never reaches an exponential growth stage. This is because the biodegradation rates of oil droplets not only depend on substrate concentration, but also on the availability of water-oil interface area. In the cases for the initial oil concentration of 0.4 and 4.0 mg/L, microbes grow without limitation at the beginning. However, at later times, the size of oil droplets decreases, which also reduce the amount of water-oil interface. As such, the cell density at the water-oil interface eventually reaches its capacity (1 cell/μm^2^) and is limited by the availability of the interface.

### Effect of initial microbe concentration

**Figure 4:**
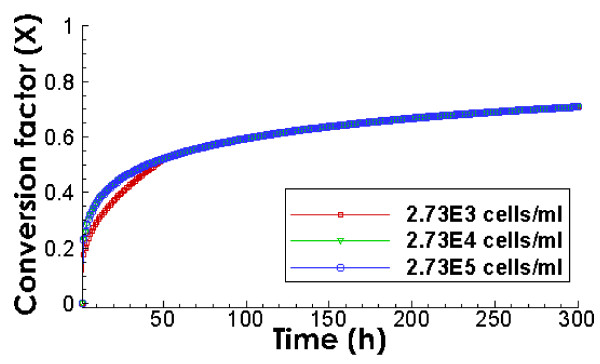
**Predicted evolution of the conversion factor X of dispersed oil droplets with different initial microbial concentrations.** The initial oil concentration is 0.4 mg/L, the mean diameter of oil droplets is 100 μm, and the CV value is 1.8.

Figure 4 shows the biodegradation of 0.4 mg/L of spilled oil in the form of oil droplets with initial microbe concentrations of 2.73 × 10^3^, 2.73 × 10^4^, and 2.73 × 10^5^ cells/ml. The comparison indicates that the initial concentrations of 2.73 × 10^3^ cells/ml would suffice to trigger the biodegradation of oil droplets at concentrations as low as 0.4 mg/L. Microbial concentrations higher than 2.73 × 10^4^ cells/ml would not increase the biodegradation of oil droplets substantially because the oil droplets surface is fully occupied by microbes (1 cell/μm^2^). This is reflected by the same biodegradation rates at 2.73 × 10^4^ and 2.73 × 10^5^ cells/ml. This is very different from the biodegradation of dissolved oil shown in Figure 2B1 where the amount of initial microbial concentration has a significant impact on the time scale of biodegradation.

### Effect of oil droplet size distribution

**Figure 5:**
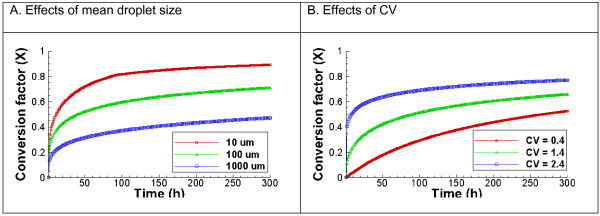
**Predicted effect of oil droplet size distribution on biodegradation kinetics.** The initial concentration of oil and microbes is 0.4 mg/L and 2.73 × 10^4^ cells/ml, respectively. Left: Three different mean diameters and the same coefficient of variation (CV) of 1.8; Right: Three CV values and the same mean diameter of 100 μm. Smaller mean droplet sizes and larger CV leads to larger water-oil-microbe contact and therefore faster and larger extent of degradation.

Oil droplets size has been reported to vary from as low as 2.5 μm [4] to as high as 2 mm [37]. According to the particle size data reported for oil spill at the Gulf of Mexico, the mean oil droplet diameter in deep water was between 20 – 30 μm. Here we use three different mean oil droplet sizes, 10, 100, and 1000 μm, to examine the effect of oil droplet size variation on the time scale for oil conversion. Figure 5A shows that the biodegradation of small oil droplets (10 μm) is faster than that of large oil droplets because the total water-oil interface is larger with small oil droplets. Oil droplet size distribution (CV) also makes a large difference, as shown in Figure 5B. With a high variation (CV value of 2.4), it takes about 1.0 day to degrade 50% of oil. In contrast, with a CV value of 0.4, only 40% of oil is degraded within 10 days. This is because high variation values means larger percentages of small oil droplets, as shown in Figure 1, which provide larger oil-water interface and therefore leads to faster initial degradation. On the other hand, low CV values such as 0.4 indicate relatively larger percentage of large oil droplets, which provide less oil-water interface for biodegradation to occur. It is generally understood that a CV of more than 2.0 indicates a highly variable size distribution. With CV values of 1.4 and 2.4, the biodegradation rate of oil droplets with a mean diameter of 100 μm levels off faster than with a CV value of 0.4, indicating that once the oil droplet size drops to certain range, oil droplet degradation occurs very slowly.

### Effect of maximum microbial density at the water-oil interface

In this section we assess the effect of maximum microbial density at the water-oil interface. For all previous cases we assumed the maximum microbial density to be 1 cell/μm^2^ using the average diameter of bacterial cell of 1 μm. In reality, the maximum microbial density on water-oil surface may be higher than 1 cell/μm^2^, because the oil degrading microbes may have a small size (<1 μm diameter), or because they may only need to be partially in contact with the water-oil interface to effectively biodegrade oil droplets. For example, typically bacteria can form biofilms which consist of layers of bacterial cells with only the most inner layer in direct 100% contact with the interface [38]. Microscopic electron images have demonstrated that biofilms are often composed of swarms of bacteria with extracellular polysaccharides serving as the adhesive agent [39]. As an alternative, it is also possible that nanofilaments (such as bacterial pili or some other mobile devices) are used in respiration of the oil rather than requiring that a single bacterium be in constant contact with the surface [40]. In these cases, the maximum microbial density at the interface can be higher than 1 cell/μm^2^. Here we compare three cases with the maximum microbial densities of 1, 5, and 10 cell/μm^2^ and with the initial concentrations of oil degrading microbes and oil droplets of 2.73 × 10^3^ cells/ml and 0.4 mg/L, respectively. Results revealed that at these typical oil and microbial concentration levels, higher maximum microbial density at the interface can result in higher rates of biodegradation only if the mean diameter of oil droplets is larger than 100 μm.

**Figure 6:**
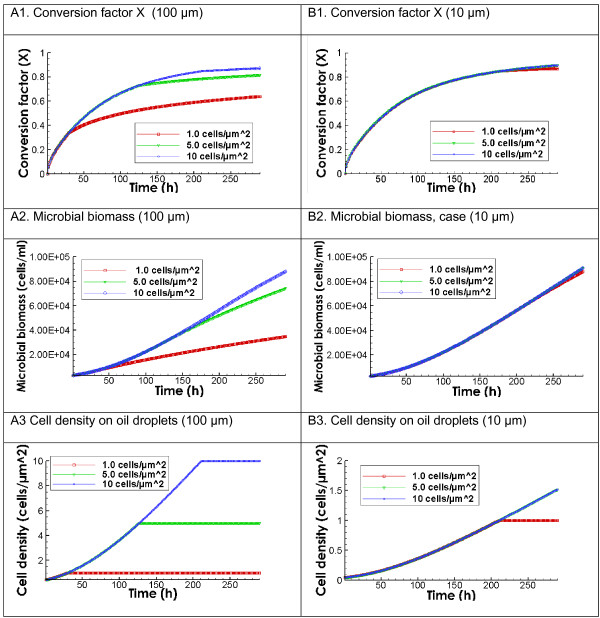
**Predicted evolution of conversion factor X, total microbial biomass, and cell density with different maximum microbial densities at the water-oil interface and with different mean oil droplet size.** Left column: Effects of maximum cell density with the oil droplet mean diameter of 100 μm. Right column: Effects of maximum cell density with the oil droplet mean diameter of 10 μm. Initial microbial and oil concentrations are 2.73 × 10^3^ cells/ml and 0.4 mg/L, respectively. The value of CV is 1.4.

Figure 6 compares the conversion factor X and microbial growth with mean oil droplet diameters of 100 (left column) and 100 μm (right column). With the mean diameter of 100 μm, the biodegradation rate increases with increasing the maximum microbial density at the water-oil interface. With other parameters being the same, the conversion factor in the 1.0 cell/μm^2^ reaches approximately 60%, while in the other two case the conversion reaches close to 80%. In addition, the difference in conversion between 1.0 and 5.0 cell/μm^2^ cases is much larger than that between 5.0 and 10.0 cell/μm^2^. This indicates that once the maximum microbial density is reached at the surface (Figure 6A3), further increasing the density will not make a difference. Correspondingly, the microbial biomass growth figure shows the much larger increase in the 5.0 and 10.0 cell/μm^2^ cases than in the 1.0 cell/μm^2^ case, indicating the difference is caused by the biomass growth. With the mean diameter of 10 μm, there is almost no difference among the three cases. This is because there is already much larger total water-oil interface area that is sufficient for the growth of bacteria than in the case of 100 μm. As a result, the increase in maximum microbial density does not make a difference in biomass growth rates and in biodegradation rates. This comparison has interesting implications. Essentially, it shows that if the amount of microbe-water-oil interface area is sufficiently large and does not limit microbial growth, the maximum microbial density does not make a difference (Figure 6B3). However, if the amount of water-oil interface area is relatively small and limits the biomass growth, the maximum microbial density makes a large difference (Figure 6A3). For the oil spill case, because the mean diameter is in the range of 20 ~ 30 μm, we expect that the increase in maximum microbial density will not make a large difference and that the prediction using 1.0 cell/μm^2^ is a good approximation.

### Effects of chemical composition

Oil is in general a complicated mixture of various types of organic chemicals. For the oil leaked in the Gulf of Mexico, it has been reported that the majority of the oil (approximately 80%) are alkanes, while the rest being other types of chemicals such as BTEX and PAHs [41,42]. Although alkanes are much easier to degrade, chemicals such as PAHs and BTEX are recalcitrant. These chemicals are also more toxic and carcinogenic. During the biodegradation the easily biodegradable chemicals will be depleted first, which leaves the recalcitrant components in the oil droplets. As such, it is important to evaluate the biodegradation kinetics of the oil droplets of different chemical composition.

Here we compare the degradation kinetics of three different chemical groups: alkanes, BTEX, and PAH. For alkanes, μ_max_ and K_s_ values of 0.6 h^-1^ and 86.0 mg/L were used based on literature values for heneicosane [17]. For BTEX, a μ_max_ value of 0.32 h^-1^ and a K_s_ value of 129.2 mg/L was used based on averaged values from literature data [16,43-47]. For PAHs, μ_max_ and K_s_ values of 0.053 h^-1^ and 28.65 mg/L were used, respectively [32,48]. From the initial and final concentrations reported for the biodegradation of oil spilled in the Gulf of Mexico [7], we calculated the yield coefficient to be equeal to1.25 × 10^8^ cells/mg-oil. This yield coefficient was assumed to be the same for alkenes, BTEX, and PAHs.

**Figure 7:**
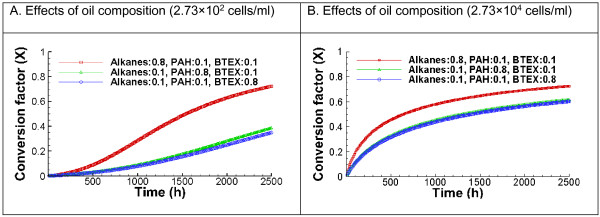
**Predicted effect of oil composition on the biodegradation kinetics.** Left: the initial microbial concentration is 2.73 × 10^**2**^ cells/ml; Right: the initial microbial concentration is 2.73 × 10^4^ cells/ml. Oil is composed of alkanes (heneicosane), BETX, and PAHs. The mean diameter of oil droplets is 100 μm, oil concentration is 0.4 mg/L, and the value of CV is 0.8.

Figure 7 shows that degradation rates differ for the oil droplets with varying chemical compositions with an initial microbial concentrations of 2.73 × 10^2^ and 2.73 × 10^4^ cells/ml. Note that for all cases, the mole fraction of some PAH compounds such as anthracene has exceeded its oil solubility of approximately 0.017 [49]. As such, some of the PAHs are in solid form in the oil droplets. It is assumed that the dissolved PAH in the oil droplets will be biodegraded first and that the further dissolution of solid PAH in the oil droplets is not rate-limiting compared to the biodegradation. This is largely true because the degradation takes thousands of hours, while the PAH dissolution into water typically occur at the time scale of hours [50]. With 2.73 × 10^2^ cells/ml, oil made up of 10% of BTEX and 10% of PAHs has been degraded for approximately only 30% after 1000 days, while the oil made up of 80% of alkanes has been degraded for more than 60%. At an initial microbial concentration of 2.73 × 10^4^ cells/ml, the biodegradation starts faster and almost stops once the cell density on the water-oil interface reaches its capacity (1 cell/μm^2^). This is similar to our previous observations in other cases. The effects of the chemical composition are larger in the case with the lower initial microbial concentration of 2.73 × 10^2^ cells/ml.

### Biodegradation of dissolved oil vs. oil droplets

**Figure 8:**
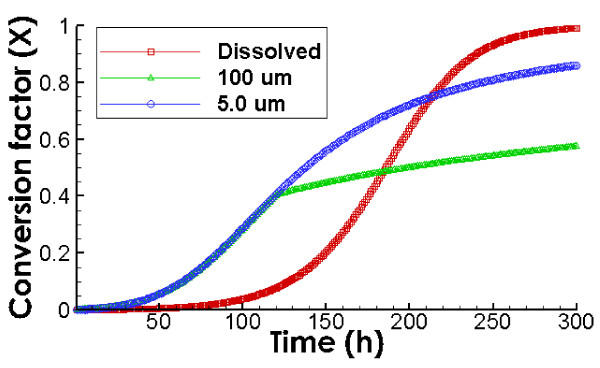
**Comparison of biodegradation kinetics for oil droplets vs. dissolved oil.** Initial microbial and oil concentrations are 2.73 × 10^2^ cells/ml and 0.4 mg/L, respectively. The value of CV is 0.8.

Figure 8 compares the predicted biodegradation kinetics by the shrinking oil droplet model and by the pseudo-component model for dissolved oil at a concentration of 0.4 mg/L. This figure clearly shows the difference in degradation kinetics of oil droplets and dissolved oil. The biodegradation rates for oil droplets and dissolved oil are both controlled by the availability of oil degrading microbes during the initial stages with low initial concentrations of microbes (2.73 × 10^2^ cells/ml). Under this condition, the biodegradation rate of oil droplets is faster than with dissolved oil because all new formed microbes accumulates on the surface of oil droplets and accelerates the biodegradation reaction, whereas new formed microbes with dissolved oil remains dispersed in the bulk fluid.

The biodegradation of 5.0 μm size starts at the same rate of 100 μm size because there is sufficient oil-water interface at the beginning. However, over time the degradation rate of oil droplets of 100 μm size levels off at a conversion factor of approximately 50% due to the lack of interface, while the biodegradation of oil droplets with 5 μm continue to increase up to 80% of complete biodegradation due to the larger available interface. For the dissolved oil, the degradation rates are low at the beginning but increase quickly over time. It eventually ends up having 100% conversion factor that is higher than that of the oil droplets, because it is not limited by the availability of the oil-water interface.

## Conclusions

The Deepwater spill in the Gulf of Mexico led to the formation of large subsurface plumes of oil droplets within several miles of the wellhead [51]. This study formulated a new mathematical model and provided a framework to describe the biodegradation of spilled oil with complex chemical compositions in the form of oil droplets. We applied the model under conditions relevant to marine oil spills and estimated the time scale of oil droplets biodegradation. Our results suggest that under conditions relevant to marine oil spills where oil concentrations are lower than 1 mg/L and background bacteria concentrations are lower than 2.73 × 10^4^ cells/ml, degradation of dispersed oil droplets with a mean diameter lower than 100 μm typically occur in two stages. The first stage is governed by the activity of oil degrading microbes reflected by high biodegradation rates. The second stage is governed by the water-oil interface availability where the oil droplets are susceptible to slower biodegradation. This is very different from the degradation of dissolved oil with no interface limitation and therefore can reach a much higher conversion factor. Compared to the dissolved oil, degradation rates of oil droplets are typically higher in early stages and slow down quickly in the second stage, resulting in a much lower ultimate conversion factor within much longer time duration.

Because water-oil interfaces play a key role in determining the oil droplet degradation, any factors that can lead to increase in water-oil interface increase the degradation rates. For example, the rates and extent of degradation are larger for oil droplets with smaller mean diameter and smaller coefficient of variation values because the oil-water surface is larger in these cases. This is consistent with observations regarding the life time of oil droplets. Venosa and Holder [10] suggested that different oil droplet size distributions that might have resulted from the addition of different types of chemical dispersants (Corexit 9500 and JD2000) can explain why biodegradation rate of the oil treated with JD2000 was several-fold higher than the biodegradation rate of the oil treated with Corexit 9500. The fact that the amount of “interface” among different phases or zones plays a key role in determining overall reaction rates is also similar to observations in other reaction systems, including mineral dissolution and microbe-mediated redox reactions in subsurface environments [52-55]. Another interesting observation is that initial bulk microbial concentration and maximum microbial density on water-oil interface have relatively smaller effects compared to dissolved oil. Besides the size distribution of oil droplets, initial oil concentrations also have an important role in determining the time scale for oil degradation.

The developed model will be useful for evaluating different remediation strategies after spill under marine environments and for predicting the timing and exposure risk of associated spills. This work provides the basis for future experimental work to evaluate the model and demonstrate its utility. This model can be incorporated into reactive transport models to explicitly evaluate the transport and fate of spilled oil in both dissolved and oil droplet form. A natural next step is to couple flow and transport processes with combined biodegradation of dissolved and dispersed oil droplets to explicitly simulate the evolution of oil composition with time to more accurately represent what occurs after the oil spill. This type of simulations should be done on case by case bases using field data on the fraction of dissolved and oil droplets, oil droplet size distribution, and initial oil degrading microbial concentrations.

It is also important to understand the results of this work in the context of the model limitations and assumption. Here we focus on the biodegradation itself without considering other processes. In the undersea or other natural environments, oil degradation typically occurs together with other processes such as flow and transport. Therefore it can be affected by these processes as well. Although we used averaged kinetic parameters to represent degradation of pseudo-compounds in each simulation, in reality the specific rate coefficient has a transient nature because the composition of oil droplets changes over time. With easily degradable compounds being transformed, the fraction of recalcitrant chemicals, such as PAH, will increase within the oil droplets of decreasing size. As such, it could take a longer time for the oil droplets to be completely degraded.

## Competing interests

The authors declare that they have no competing interests.

## Authors’ contributions

JV, LL, and SSH discussed and initiated the idea about developing a model for the biodegradation of oil droplets relative to the Gulf of Mexico oil spill. JV and LL discussed and carried out the detailed derivation and equation solving. JV and LL drafted the manuscript. SSH provided assistance in editing and finalizing the manuscript. All authors read and approved the final manuscript and contributed to the revision.
